# Longitudinal changes in peritoneal solute transport rate and the impact of lower glucose degradation product glucose dialysates

**DOI:** 10.1111/1744-9987.70012

**Published:** 2025-03-24

**Authors:** Andrew Davenport

**Affiliations:** ^1^ UCL Centre for Kidney and Bladder Health, Royal Free Hospital University College London London UK

**Keywords:** bioimpedance, end stage kidney disease, glucose degradation products, icodextrin, peritoneal dialysis, peritoneal solute transport rate

## Abstract

**Introduction:**

Peritoneal solute transfer rates (PSTR) are reported to increase with time. Changes in PSTR were reviewed in long‐term peritoneal dialysis (PD) patients to determine whether lower glucose degradation products (low GDP) dialysates prevented an increase in PSTR.

**Methods:**

PSTR was determined with a 4‐h peritoneal equilibrium test with a 2.0 L 22.7 g/L glucose dialysate.

**Results:**

One hundred twenty‐three PD patients treated for ≥4 years, 47.2% male, age 61 ± 16 years, 31.7% diabetic. Initially, 15.6% were treated with low GDP dialysates, which rose to 44.7% at 6 years. Creatinine PSTR increased with standard glucose dialysates (0.72 ± 0.1 at Year 3 to 0.79 ± 0.1 Year 5 and 0.82 ± 0.1 Year 6, *p* < 0.05), whereas PSTR was stable and lower with low GDP dialysates (0.71 ± 0.1, 0.65 ± 0.1, 0.68 ± 0.1); *p* < 0.001 for Years 5 and 6.

**Conclusion:**

Exposure to standard glucose dialysates resulted in faster peritoneal solute transfer rates over time, whereas peritoneal solute transfer rates appeared more stable with lower glucose degradation products dialysates.

## 1 INTRODUCTION

Although world‐wide more patients with end‐stage kidney disease (ESKD) are treated with hemodialysis, a substantial number of around 300 000 are treated with peritoneal dialysis (PD). However, technique survival has consistently been reported to be lower than that for hemodialysis [1]. Historically, peritoneal dialysates have used hypertonic glucose solutions to obtain ultrafiltration and control hypervolaemia. As dialysates are heat sterilized, heating hypertonic glucose solutions leads to the production of a range of glucose degradation products (GDPs). Exposure to hypertonic glucose dialysates has been reported to be associated with changes to the vasculature, interstitium, and mesothelial lining of the peritoneal cavity [2]. To reduce these potential deleterious effects, hypertonic glucose dialysates containing lower GDPs have been developed, along with the introduction of oncotic alternatives to glucose.

The three‐pore model has been traditionally used to describe peritoneal mass transport [3], based on the concept of ultra small, small, and large‐sized pores. When hypertonic glucose and icodextrin dialysates are first instilled into the abdomen, there is an initial difference in the movement of water and sodium, as water predominantly moves through aquaporin channels (ultra small pores) [4], whereas sodium requires primary or secondary active transport (small pores). This result in an initial faster movement of water compared to sodium into the peritoneal cavity, and a dip in dialysate sodium. This sodium dip has been reported to be a surrogate for the osmotic conductance to glucose (OCG) and reflects the water transport properties of the peritoneum [5]. Proteins, on the other hand, move through large pores, mainly through venular large inter‐endothelial pores, but also via transcellular passage, with movement varying for individual proteins according to size and charge [6].

Several observational studies reported that peritoneal membrane transport changed over time, with patients developing faster peritoneal solute transporter rates (PSTR) based on peritoneal creatinine clearance, assessed with a standard 4‐h 2.0 L 22.7 g/L dwell (peritoneal equilibrium test [PET] test) [7, 8]. This increase in PSTR was associated with greater vasculopathy and interstitial expansion in peritoneal biopsies [2] Later reports suggested that although PSTR increased over time with standard hypertonic glucose dialysates, the PSTR stabilized in patients using the lower glucose degradation product dialysates [8].

As such, we wished to review changes in PSTR over time to determine whether there were changes in ultra small, small, or large pore transport and exposure to hypertonic glucose dialysates.

## 2 METHODS

We reviewed the records of adult patients electively starting PD under the care of an inner‐city university hospital, between January 2001 and December 2019 who had been treated by PD for 4 years or more. Patients were prescribed varying 13.6 g/L, 22.7 g/L standard glucose and icodextrin dialysates, and lower GDP dialysates (Physioneal), all supplied by Baxter Health Care (Deerfield, Illinois, USA). Patients routinely attended for their first assessment of peritoneal membrane function 2–3 months after completing PD training, and then annually or delayed by 3 months after an episode of peritonitis, or any intercurrent illness requiring hospitalization. Peritoneal membrane assessment was determined using a standard 4‐h dwell with a 2.0 L 22.7 g/L dextrose exchange (Baxter Health Care, Deerfield, Illinois, USA) [9]. To standardize the PET test, all patients were required to attend with a 22.7 g/L dextrose dialysate instilled. Fresh peritoneal dialysate and effluent bags were weighed, and the difference recorded after the effluent drained. PSTR was categorized according to European clinical practice guidelines [10]; 4‐h dialysate/plasma creatinine ratio fast >0.8 and <0.55–0.6 slow. In addition, 24‐h urine collections were analyzed to determine residual kidney function, which was reported as the mean of urea and creatinine clearances. Normalized nitrogen appearance (nPNA) was estimated from urinary and spent 24‐h peritoneal dialysate urea measurements [11]. PD adequacy was determined by standard measurements, and daily peritoneal glucose absorption and sodium removal calculated as the difference in the total amount of sodium and glucose instilled in fresh dialysates and that measured in drained effluent [12, 13]. Multi‐frequency bioelectrical impedance assessments (MFBIA) were carried out after completing peritoneal membrane testing [14], after patients had drained out peritoneal dialysate and emptied the bladder [15]. MFBIAs were performed in a standardized manner (InBody 720, Seoul, South Korea) as part of established routine clinical care [16]. Bioimpedance equipment was regularly serviced and calibrated. Relative extracellular water (ECW) over hydration was expressed as the ratio of ECW to total body water (TBW) and height [17]. Patients with amputations, and those unable to stand were excluded from the study.

Serum biochemistry samples were analyzed using a standard multi‐channel biochemical analyzer, using the bromocresol green method for albumin determination, C reactive protein (CRP), and hemoglobin samples by standard methodology (XE‐2100 Sysmex Corporation, Kobe, Japan). N‐terminal probrain natriuretic peptide (NTproBNP) and total protein by the benzethonium chloride method were measured with an enzyme‐linked immunosorbent assay (ELISA) (Roche Cobra, Roche diagnostics, Lewes, UK) [18, 19]. Dialysate creatinine was measured using a kinetic enzymatic method to prevent glucose interference (P module analyzer, Roche Integra, Roche diagnostics, Lewes, UK) [20].

Patient‐related data was obtained from hospital computerized records and co‐morbidity assessed using the Stoke‐Davies grading scales [21], and frailty according to the Canadian Society of Gerontology scale [22].

### 2.1 Statistical methods

Categorical data are presented as numbers (%) and continuous data as mean ± standard deviation for normally distributed data or median (interquartile range) for non‐parametric data. Standard statistical tests were used to analyze data, including Chi square (*χ* ^2^) analysis for categorical data, and analysis of variance (Anova) to compare numerical data between groups and paired analyses with time with appropriate corrections made for multiple testing (Tukey and Games Howell). Statistical analysis was carried out using Prism 10.4 (Graph Pad, San Diego, USA) and SPSS29 (IBM, Armonk, NY, USA). Statistical significance was taken at *p* < 0.05.

### 2.2 Ethical approval

This retrospective audit followed and complied with United Kingdom National Research Ethics Service clinical audit and service development, with the audit of clinical service registered and individual patient consent waived by the United Kingdom National Research Ethics Service. In accordance with regulatory guidelines, all patient data was appropriately anonymized.

## 3 RESULTS

One hundred and twenty‐three patients from a total of 868 (14.2%) had been treated by PD for 4 years, 75 (8.6%) for ≥5 years, and 38 (4.4%) for ≥6 years. The cohort comprised 58 men (47.2%), mean age 61 ± 16 years, 39 diabetic (31.7%) patients, and the main ethnic groups were white 55 (44.7%), Asian 35 (28.5%), and African‐Afro‐Caribbean 31 (25.2%).

Over time, the number of patients treated by automated PD cyclers with a dry day (APD) decreased, whereas those treated with cyclers and a day exchange increased, as did the use of lower GDP glucose dialysates (Figure S1), all *p* < 0.001. Similarly, as residual kidney function declined, the amount of hypertonic (22.7 g/L) glucose dialysates and icodextrin prescribed increased, resulting in increasing peritoneal ultrafiltration and peritoneal glucose absorption (Table 1). Estimated dietary protein intake was lower in Years 5 and 6, but there was no significant change in either percentage body fat or lean body mass (Table 2).

**TABLE 1:** From the first peritoneal equilibrium test (PET) and then annually. Months of peritoneal dialysis (vintage mo), continuous peritoneal dialysis (CAPD), automated peritoneal dialysis dry day (APD), automated peritoneal dialysis wet day continuous cycling peritoneal dialysis (CCPD), volume icodextrin (Ico), 22.7 g/L dextrose (22.7), % prescribed low glucose degradation products glucose dialysates (low GDP), weekly urea clearance (Kt/V), urine output (UO), urinary creatinine clearance (CrCl) mL/min, 24 h. Peritoneal ultrafiltration (24 h UF) L/day, peritoneal glucose absorption (Gluc Abs) mmol/day, normalized estimated protein nitrogen appearance (nPNA) g/kg/day, PET transporter classification fast, average, and slow (10), 4 h ratio creatinine (D/Pcreat), sodium (D/P Na), phosphate (D/PPi), protein D/Pprot, ultrafiltration volume (PET UF) mL. Data expressed as integer, mean ± SD, or median (interquartile range). *p < 0.05, **p < 0.01, ***p < 0.001 vs. first PET.

	First PET	12 mo	24 mo	36 mo	48 mo	60 mo	72 mo
Patients	123	123	123	123	123	78	38
Vintage mo	2 (2–3)	14 (12–15)***	25 (23–27)***	37 (35–38)***	49 (7–50)***	60 (57–62)***	72 (70–73)***
Frailty	3.5 ± 1.3	3.5 ± 1.1	3.5 ± 1.3	3.9 ± 1.1	4.0 ± 1.4	4.1 ± 1.2	4.3 ± 1.2
Comorbidity	1.0 ± 0.9	1.1 ± 1.0	1.1 ± 0.9	1.2 ± 1.0	1.2 ± 1.0	1.3 ± 1.0	1.6 ± 1.1*
Ico (L/day)	1.1 ± 1.0	1.5 ± 1.0	1.6 ± 1.0*	1.6 ± 1.0***	1.7 ± 1.0***	1.7 ± 1.0***	1.8 ± 1.0*
22.7 L/day	1.6 ± 3.4	2.4 ± 3.7	3.2 ± 4.5*	3.9 ± 4.9***	4.9 ± 5.3***	5.1 ± 5.2***	5.9 ± 5.6**
Weekly (Kt/V)	2.81 ± 1.24	2.29 ± 0.84***	2.18 ± 0.86***	2.12 ± 0.76**	1.91 ± 0.66**	1.91 ± 0.6**	2.02 ± 1.13**
UO (L/day)	1.2 ± 0.8	0.9 ± 0.7	0.8 ± 0.7**	0.8 ± 0.7***	0.6 ± 0.6***	0.6 ± 0.6***	0.5 ± 0.6***
CrCl (mL/min)	8.7 ± 6.8	6.0 ± 5.4*	4.7 ± 4.7***	3.6 ± 3.6***	2.5 ± 3.2***	2.1 ± 2.6***	2.3 ± 5.1***
24 h UF mL	552 ± 509	693 ± 561	743 ± 579	852 ± 611***	857 ± 637***	893 ± 526***	967 ± 545***
24 h Gluc Abs	141 ± 135	137 ± 153	162 ± 160	190 ± 170	216 ± 201*	221 ± 191*	215 ± 203*
nPNA (g/kg/day)	0.96 ± 0.28	0.92 ± 0.23	0.91 ± 0.27	0.94 ± 0.28	0.88 ± 0.27	0.85 ± 0.22*	0.86 ± 0.22*
Slow %	19.5	15.1	13.2	16.3	14.6	14.1	7.9
Average %	52.0	52.1	62.0	60.5	53.7	65.4	60.5
Fast %	28.5	32.8	16.5	23.3	31.7	29.5	31.6
4hD/Pcreat	0.72 ± 0.14	0.73 ± 0.13	0.72 ± 0.13	0.73 ± 0.13	0.72 ± 0.13	0.74 ± 0.13	0.76 ± 0.12
4hD/P Na	0.929 ± 0.035	0.934 + 0.031	0.946 + 0.031	0.943 + 0.032*	0.946 + 0.031**	0.943 + 0.031	0.947 + 0.031*
4hD/PPi	0.63 ± 0.23	0.71 ± 0.72	0.63 ± 0.14	0.64 ± 0.14	0.63 ± 0.14	0.63 ± 0.16	0.67 ± 0.14
4hD/Do Gluc	0.33 ± 0.08	0.34 ± 0.09	0.33 ± 0.08	0.34 ± 0.08	0.34 ± 0.08	0.34 ± 0.09	0.33 ± 0.09
4hD/PProtein	0.93 ± 0.58	0.81 ± 0.39	0.84 ± 0.15	0.8 ± 0.47	0.76 ± 0.36	1.0 ± 1.8	0.79 ± 0.41
PET UF (mL)	241 ± 213	274 ± 254	285 ± 299	275 ± 321	260 ± 223	208 ± 213	163 ± 233

**TABLE 2:** Results of standard laboratory investigations and body composition measurements from bioimpedance measurements from the first peritoneal equilibrium test (PET) and then annually. Hemoglobin (Hb), C reactive protein (CRP), glycated hemoglobin (HbA1c) mmol/mol, N‐terminal probrain natriuretic peptide (NTproBNP) pg/mL, serum total protein (protein), body mass index (BMI), soft lean muscle mass index (SLMI). Appendicular lean mass index (ALMI), % body fat mass (BFM), extracellular water (ECW), total body water (TBW), height (ht), number of classes of blood pressure medications (BP meds). Data expressed as mean ± SD or median (interquartile range). *p < 0.05, **p < 0.01, ***p < 0.001 vs. first PET.

	First PET	12 mo	24 mo	36 mo	48 mo	60 mo	72 mo
Hb (g/L)	111.5 ± 14.8	109.7 ± 16.4	110.1 ± 14	107.2 ± 12.8	108.4 ± 14.6	108.2 ± 14.5	103.2 ± 13.1
Urea (mmol/L)	19 ± 5.4	19.7 ± 5.3	20.0 ± 6.0	19.7 ± 6.3	19.2 ± 6.0	17.9 ± 6.1	18.3 ± 4.6
Creatinine (umol/L)	613 ± 265	692 ± 287	784 ± 324**	798 ± 301***	854 ± 297***	7890 V275***	762 ± 258***
Albumin (g/L)	37.5 ± 5.0	38.8 ± 4.1**	38.5 ± 3.9	38.5 ± 4.1	37.9 ± 4.2	36.8 ± 4.2	35.6 ± 4.7**
CRP (mg/L)	3 (1–7)	3 (2–7)	3 (1–7)	4 (1–10)	4 (1–11)	4 (1–12)	4.5 (2–13)
Glucose (mmol/L)	6.7 ± 3.6	7.1 ± 4.7	7.3 ± 4.1	7.4 ± 4.9	7.3 ± 3.9	7. ±4.5	8.2 ± 4.6
HBA1c (mmol/mol)	37 (33–44)	38 (33–45)	38 (33–48)	38 (33–46)	40 (35–47)	40 (34–51)	43 (39–48)
NTproBNP (pg/mL)	1729 (753–4647)	1844 (814–4490)	2689 (909–7205)	2843 (1446–7628)	3930 (1245–12 685)***	4330 (1970–1704)***	8931 (4216–32 204)***
Sodium (mmol/L)	138 ± 4	137 ± 4**	137 ± 4	136 ± 4**	136 ± 4***	135 ± 4**	135 ± 4***
Protein (g/L)	67 ± 6.	67 ± 7	67 ± 7	67 ± 6	66 ± 7	65 ± 6	63 ± 6*
Weight (kg)	70.1 ± 16.6	71.0 ± 16.4	71.5 ± 16.4	69.8 ± 14.6	69.5 ± 15.4	68.3 ± 14.9	66.0 ± 14.2
BMI (kg/m^2^)	25.8 ± 5.3	26.1 ± 4.9	26.4 ± 4.8	25.8 ± 4.4	25.5 ± 4.4	25.0 ± 4.3	24.7 ± 4.8
SLMI (kg/m^2^)	9.8 ± 2.8	9.3 ± 1.5	9.3 ± 1.6	9.2 ± 1.5	9.1 ± 1.4	9.0 ± 1.3	9.2 ± 1.7
ALMI (kg/m^2^)	7.4 ± 1.9	7.0 ± 1.6	7.2 ± 1.8	7.5 ± 4.4	7.0 ± 1.4	6.9 ± 1.2	6.9 ± 1.5
BFM (%)	30.1 ± 14.1	32 ± 10	33 ± 10.3	32.3 ± 11.2	32.7 ± 10.6	31.5 ± 10.3	29.0 ± 9.9
ECW/TBW (%)	39.6 ± 1.3	39.3 ± 1.2*	39.7 ± 1.6	39.7 ± 2.5	39.8 ± 1.8	40.0 ± 1.3	40.2 ± 1.4
ECW/ht (L/m)	8.7 ± 2.3	8.3 ± 1.6***	8.3 ± 1.6	8.3 ± 1.6	8.1 ± 1.4	8.3 ± 1.4	8.5 ± 1.5
BP meds	1 (1–2)	1 (0–2)	1 (0–2)	1 (0–2)**	1 (0–2)	0 (0–1)**	1 (0–1)*

N‐terminal probrain natriuretic peptide (NTproBNP) concentrations increased over time, whereas serum sodium declined (Table 2). Serum albumin was highest at 12 months and lowest after 6 years. Estimates of volume overload, ECW/TBW and ECW/height (ECW/ht) were lowest at 12 months, and ECW/TBW was greater at Years 4–6 compared to that at 12 months (all *p* < 0.001). ECW/ht was greater at Year 6 compared to Years 3 and 4, *p* < 0.05. However, the number of different classes of antihypertensive medications prescribed decreased with time. Overall, when taking into account total daily peritoneal ultrafiltration and urinary volumes, the greater 24‐h volume removed was associated with both a lower NTproBNP (*r* = −0.27, *p* < 0.001) and a lower ECW/TBW ratio (*r* = −0.12, *p* = 0.008).

Over time there was no significant increase in the PET 4‐h dialysate to plasma creatinine ratios or the number of fast peritoneal transporters (Table 1). Similarly, there was no change in the ratios of 4‐h dialysate to plasma phosphate or total protein. However, there was an increase in the ratio of dialysate to plasma sodium at both 4 and 2 h, so there was less sodium dip over time (Table 1 and Figures 2 and S2).

We then compared patients who had either started with low GDP glucose dialysates or switched from standard glucose to low GDP glucose dialysates, and over time the proportion of patients using lower GDP dialysates increased steadily from 15.6% to 18.6% after 12 months, and 21% after 24 months to 44.7% at 6 years. In total, there were 125 episodes of PD peritonitis, 96 during treatment with standard glucose dialysates and 29 with lower GDP glucose dialysates (*χ* ^2^ = 0.5, *p* = 0.5).

**FIGURE 1:**
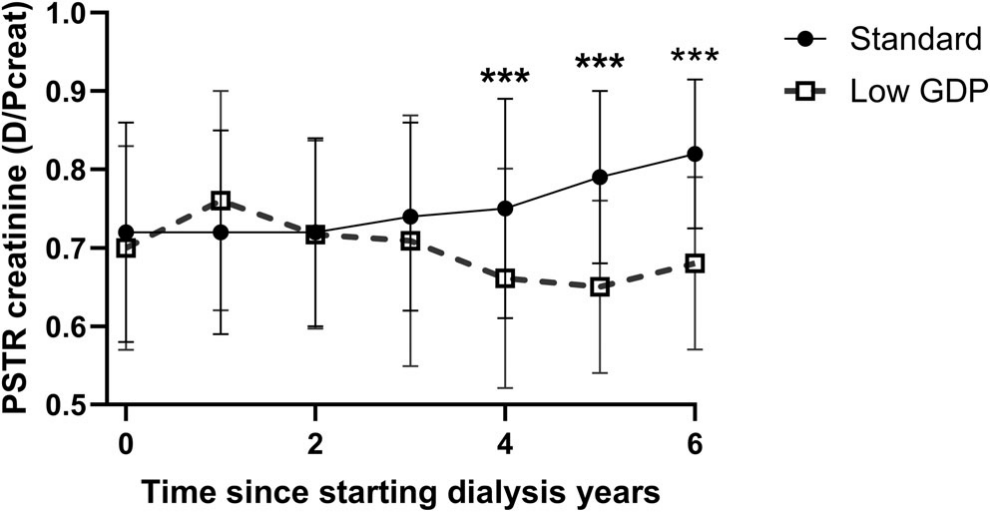
The 4‐h effluent dialysate to plasma serum creatinine (D4h D/P Creat) after a 4‐h 2.0 L 22.7 g/L comparing the result for the first assessment of peritoneal membrane function (peritoneal equilibrium test [PET]) over time. Median with interquartile and 10%–90% ranges. ***p < 0.001 vs. first PET. GDP, glucose degradation products.

**FIGURE 2:**
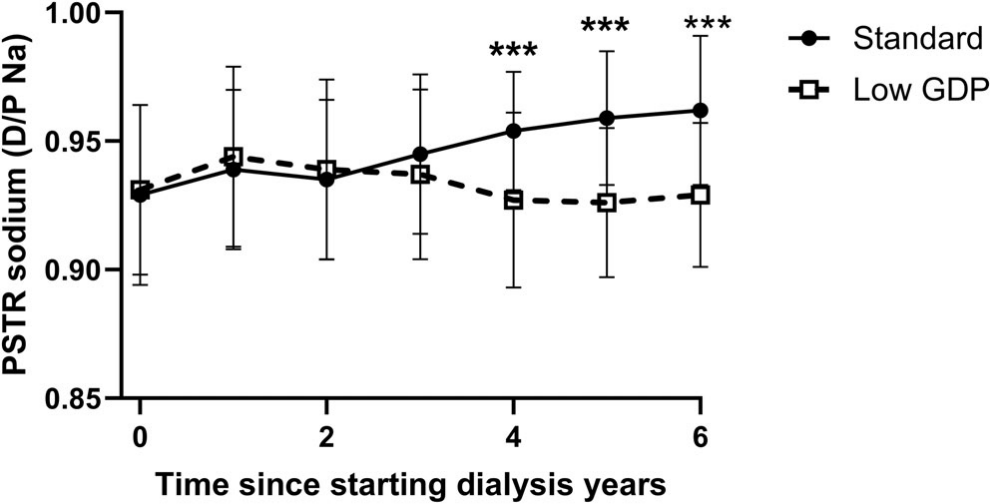
The 4‐h effluent dialysate to plasma serum sodium (D4h D/P Na) after a 4‐h 2.0 L 22.7 g/L exchange comparing the results for the first assessment of peritoneal membrane function (peritoneal equilibrium test [PET]) over time. Median with interquartile and 10%–90% ranges. ***p < 0.001 vs. first PET. GDP, glucose degradation products.

Whereas there was an increase in PSTR in those prescribed standard glucose dialysates over time, there was no significant increase in those prescribed lower GDP glucose dialysates (Table 3), and patients prescribed standard glucose dialysates had faster PSTR for creatinine after 4 years (Figure 1). Similarly, the 4‐h D/P sodium increased in the standard glucose dialysate group, and the sodium dip was significantly different between groups (Figure 2). Although there was a trend for a lower serum sodium and albumin and higher ratios of ECW to both TBW and height, these differences were generally not statistically significant (Table 3). Urine output was initially higher in the standard glucose dialysate group at 12 months (927 [460–1504] vs. 624 [226–1276], *p* < 0.05, but then lower at 3 years, but not different at other times; Table 3). Although there was no difference in the volumes of icodextrin prescribed, the standard glucose dialysate group was initially prescribed less hypertonic glucose (22.7 g/L) at the first PET test (0 [0–0] vs. 1.0 [0–4.6] L, *p* < 0.01) and at 12 months (0 [0–4] vs. 3.3 [0–8.7] L, *p* < 0.01), but thereafter volumes of hypertonic glucose used did not differ between groups.

**TABLE 3:** Results of peritoneal solute transport rates from 4‐h peritoneal equilibrium test (PET) using 22.7 g/L glucose dialysate. Comparison of dialysate to plasma (D/P) from Years 3 to 6 in patients who were prescribed standard glucose‐containing dialysates and those prescribed low glucose degradation products (GDP) glucose dialysates. Urine output (UO). Data presented as %, mean ± standard deviation, or median and interquartile range. *p < 0.05, **p < 0.01, ***p < 0.001 standard vs. low GDP dialysates, and ^+^ *p* < 0.05, ^+++^ *p* < 0.05 vs. first PET D/Pcreat for both groups.

	Year 3	Year 4	Year 5	Year 6
Low GDP	27.6%	32.2%	39.7%	44.7%
Standard 4 h D/P creatinine	0.74 ± 0.12	0.75 ± 0.12^+++^	0.79 ± 0.09^+++^	0.82 ± 0.10^+++^
Low GDP 4 h D/P Creatinine	0.71 ± 0.12	0.67 ± 0.14	0.65 ± 0.22	0.68 ± 0.20
Standard 4 h D/P Sodium	0.945 ± 0.031^+^	0.954 ± 0.023^+++^	0.954 ± 0.026^+++^	0.962 ± 0.029^+++^
Low GDP 4 h D/P Sodium	0.937 ± 0.033*	0.927 ± 0.034***	0.926 ± 0.029***	0.929 ± 0.028**
Standard serum albumin (g/L)	38.2 ± 4.0	37.3 ± 4.2	36.2 ± 4.4	34.9 ± 5.2
Low GDP serum albumin (g/L)	38.7 ± 4.0	38.8 ± 3.9	37.6 ± 3.1	36.4 ± 3.8
Standard serum sodium (mmol/L)	136 ± 4.0	135 ± 4.0	134 ± 4.0	134 ± 4.0
Low GDP serum sodium (mmol/L)	137 ± 4.0	137 ± 4.0	137 ± 4.0*	136 ± 4.0
Standard ECW/TWB%	39.9 ± 2.8	40.0 ± 1.6	40.3 ± 1.3	40.6 ± 1.5
Low GDP ECW/TBW%	3.92 ± 1.3	39.5 ± 2.1	39.5 ± 1.1*	39.9 ± 1.3
Standard ECW/ht (L/m)	8.47 ± 1.72	8.29 ± 1.53	8.41 ± 1.51	8.79 ± 1.44
Low GDP ECW/ht (L/m)	7.75 ± 1.27*	7.96 ± 1.05	8.09 ± 1.32	8.19 ± 1.45
Standard UO (L/day)	0.5 (0.03–1.0)	0.3 (0–0.78)	0.3 (0–0.82)	0.24 (0.04–0.9)
Low GDP UO (L/day)	0.92 (0.25–1.65)*	0.55 (0–1.12)	0.53 (0–0.99)	0.4 (0.01–0.75)
Standard icodextrin (L/day)	1.9 (1.3–2.0)	2.0 (1.3–2.0)	1.8 (1.0–2.0)	1.8 (1.3–2.0)
Low GDP icodextrin (L/day)	1.8 (1.0–2.0)	1.8 (1.0–2.0)	1.6 (1.3–2.0)	1.5 (1.5–2.0)
Standard 22.7 g/L glucose (L/day)	2.0 (0–8.0)	4.5 (0–10.7)	4.7 (0–9.6)	4.7 (0–9.6)
Low GDP 22.7 g/L glucose (L/day)	2.0 (0–4.7)	4.0 (0–4.9)	2.0 (0–8.4)	4.7 (2.0–12.1)

Abbreviations: ECW, extracellular water; TBW, total body water.

As the majority of patients prescribed lower GDP glucose dialysates had switched from standard to lower GDP glucose dialysates, we compared the PET test when last treated with standard dialysates to the first using lower GDP dialysates. However, there were no statistical differences in PSTR (D/P 4 h creatinine 0.71 ± 0.16 vs. 0.73 ± 0.13, 4‐h D/P sodium 0.933 ± 0.032 vs. 0.941 ± 0.031, ultrafiltration volume 200 [100–300] vs. 200 [100–400], and 2‐h D/P sodium 0.922 ± 0.03 vs. 0.932 ± 0.031).

There was no difference in the number of episodes of peritonitis when patients were treated with standard glucose dialysates and low GDP dialysates (median/patient 1 [0–2] vs. 0 [0–1], *p* = 0.35). We then analyzed the number of episodes of peritonitis in patients treated for 3 years (0 [0–1] vs. 1 [0–1]), 4 years (1 [0–2] vs. 0 [0–2]), 5 years 1 (0–1) vs. 0 (0–1), and 6 years (10–1) vs. 1 (0–3), and there was only a difference in Year 5, *p* = 0.021.

## 4 DISCUSSION

Older observational studies reporting on PSTR found that peritoneal solute clearance increased over time and that faster peritoneal transport was associated with increased risk of treatment failure [23, 24]. However, these earlier studies reported on ESKD patients treated with continuous ambulatory PD and before the introduction of automated peritoneal cyclers and icodextrin dialysates. In addition, PET tests were often not standardized, with patients attending with different peritoneal dialysates instilled immediately prior to the PET, potentially altering the glucose gradient and high glucose concentrations interfering with colorimetric creatinine assays [25]. In this more contemporaneous cohort of patients treated with both continuous ambulatory PD and automated peritoneal cyclers, with more than 100 patients treated for ≥4 years and 75 for ≥5 years, we did not observe a change or trend in PSTR over time for creatinine for the whole cohort [10]. However, compared to earlier studies [23, 24], most patients were prescribed icodextrin dialysates, and over time more patients were switched to glucose dialysates with lower GDPs, although the majority remained on standard glucose dialysates. We also measured peritoneal phosphate transport [26], and again there was no significant change in phosphate transport over time for the whole group. Similarly, in keeping with previous reports, we did not observe a change in total protein transport [27, 28]. Other small studies noted a reduction in daily peritoneal protein losses associated with a reduction in peritoneal ultrafiltration [29]. Although there appeared to be a trend in a reduction in PET ultrafiltration volumes over time, this was not significant. More recently, the sodium dip has been proposed as a surrogate for glucose conductance to overcome the inherent inaccuracies in ultrafiltration volumes [5]. Although some small studies did not note a change in sodium dip over time [29], we found that the sodium dip at 2 and 4 h for the whole cohort declined over time on PD.

As some previous reports have suggested a more stable PSTR over time with lower GDP dialysates, we compared those who were initially started with or subsequently switched to these dialysates [30]. As episodes of peritonitis could potentially affect peritoneal membrane function, we reviewed infection data, and overall, there was no significant difference in the number of episodes of peritonitis between groups. In keeping with some other recent studies [30], we noted that PSTR did not significantly increase over time for those who started with or converted to using lower GDP glucose dialysates, whereas there was a trend for an increase in creatinine PSTR with standard glucose dialysates, which became significant after 5 years. Similarly, whereas the sodium dip at 4 h did not significantly reduce with the lower GDP glucose dialysates, this reduction in the standard group became significant after 3 years. This would suggest reduced water transport in response to the glucose load [5]. Although developing faster PSTR was initially associated with an increased risk of both treatment failure and mortality, this increased risk was suggested to be reduced by the introduction of automated PD cyclers with shorter dwell times and icodextrin [31]. However, despite increasing use of PD cyclers in our cohort and icodextrin, the relative trends in serum sodium and albumin [32] and ratios of ECW to TBW and height would suggest greater fluid retention over time in those patients continuing with standard glucose dialysates [17, 33].

Due to the relatively high turnover of patients treated by PD, there are few observational reports documenting changes in PSTR over time. As such, these reports can be affected by survivor bias and changes in clinical practice over time. Although we cannot exclude any survivor bias, we maintained a standardized protocol for performing PET and bioimpedance assessments and laboratory measurements. When comparing patients prescribed lower GDP dialysates, then ideally, all our patients using lower GDP glucose dialysates would have been prescribed these from the start of PD, rather than around 50% starting with these dialysates. However, baseline demographics were similar, including co‐morbidities and clinical frailty scores, and the prescription of hypertonic glucose dialysates (22.7 g/L) was higher during the first year of treatment, and over time, there was no difference in the number of episodes of PD peritonitis between groups. As we only used lower GDP glucose dialysates from one manufacturer, our results may not equally apply to all lower GDP glucose dialysates.

There have been few studies reporting on longitudinal changes in PSTR over 5 or more years. Overall, as urine output fell, more patients were prescribed increasing volumes of hypertonic glucose and transferred to APD cyclers with a daytime fill. PSTR for creatinine, phosphate, and protein did not notably change for the whole group, although that for sodium increased, suggesting a reduction in osmotic conductance and free water clearance [5]. Comparing patients who initiated or transferred to lower GDP glucose dialysates, those who remained on standard glucose dialysates were observed to have an increased PSTR for creatinine as well as sodium, suggesting that exposure to glucose dialysates containing higher amounts of GDPs causes greater changes to peritoneal structures [34], predominantly affecting ultra small pore, but also on small pore transport. Over time, these changes in peritoneal function will increase the risk of subsequent ultrafiltration failure.

## CONFLICT OF INTEREST STATEMENT

The author has any conflicts of interest or competing interests.

## ETHICS STATEMENT

Ethics complies with UK National Research Ethics guidelines for audit and clinical service development.

## Supporting information


**Figure S1.** To demonstrate the changes in clinical practice over the time course of the report. The percentage of patients treated with continuous ambulatory peritoneal dialysis (CAPD), automated peritoneal dialysis over night with a dry day (APD), and overnight automated peritoneal dialysis with a day‐time dwell (CCPD) changed, with more patients prescribed cycler peritoneal dialysis. Similarly, the prescription of low glucose degradation glucose dialysates (low GDP) over time from the first assessment of peritoneal membrane function (PET) also increased. Compared to first PET the use of APD declined (*χ* ^2^ 24.6, *p* < 0.001), as did the prescription of low GDP glucose dialysates (*χ* ^2^ 28.9, *p* < 0.001).

**Figure S2.** Changes in the 2‐h effluent dialysate to plasma serum sodium (D2h D/P Na) ratio after 2‐h using a2.0 L 22.7 g/L exchange, comparing the result obtained with *r* the first assessment of peritoneal membrane function (PET) over time. Median with interquartile and 10%–90% ranges. **p < 0.01, ***p < 0.001 vs. first PET.

## Data Availability

Data are retained and may be available on reasonable request with all identifiers removed in keeping with current UK NHS guidelines.
