# Supplementary figures and images for: Longitudinal changes in peritoneal solute transport rate and the impact of lower glucose degradation product glucose dialysates
Source: Ther Apher Dial. 2025 Mar 24;29(3):471–8. doi: 10.1111/1744-9987.70012 (PMC12050142; doi:10.1111/1744-9987.70012)

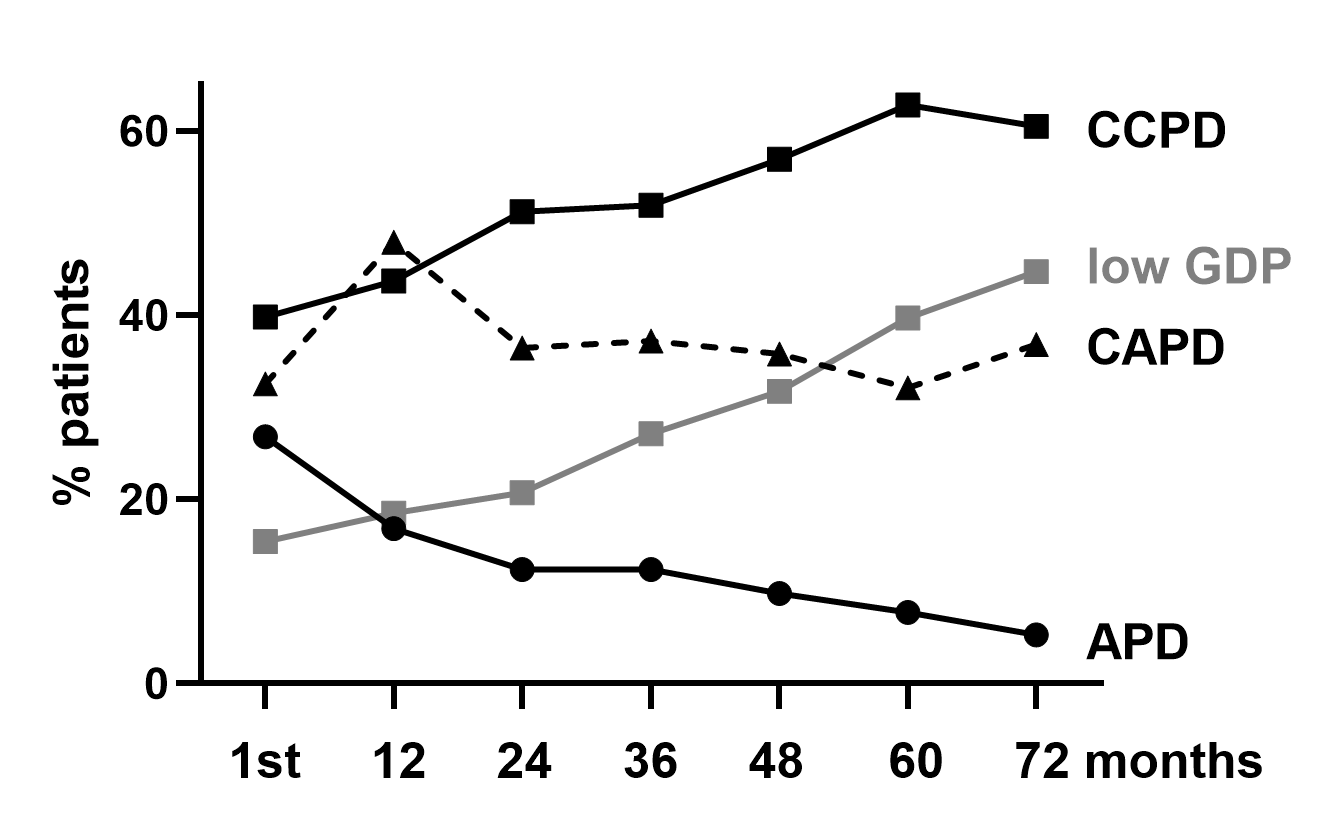

Supplement: Supplementary file 1 — Figure S1. To demonstrate the changes in clinical practice over the time course of the report. The percentage of patients treated with continuous ambulatory peritoneal dialysis (CAPD), automated peritoneal dialysis over night with a dry day (APD), and overnight automated peritoneal dialysis with a day‐time dwell (CCPD) changed, with more patients prescribed cycler peritoneal dialysis. Similarly, the prescription of low glucose degradation glucose dialysates (low GDP) over time from the first assessment of peritoneal membrane function (PET) also increased. Compared to first PET the use of APD declined (χ 2 24.6, p < 0.001), as did the prescription of low GDP glucose dialysates (χ 2 28.9, p < 0.001). [file TAP-29-471-s001.tif]

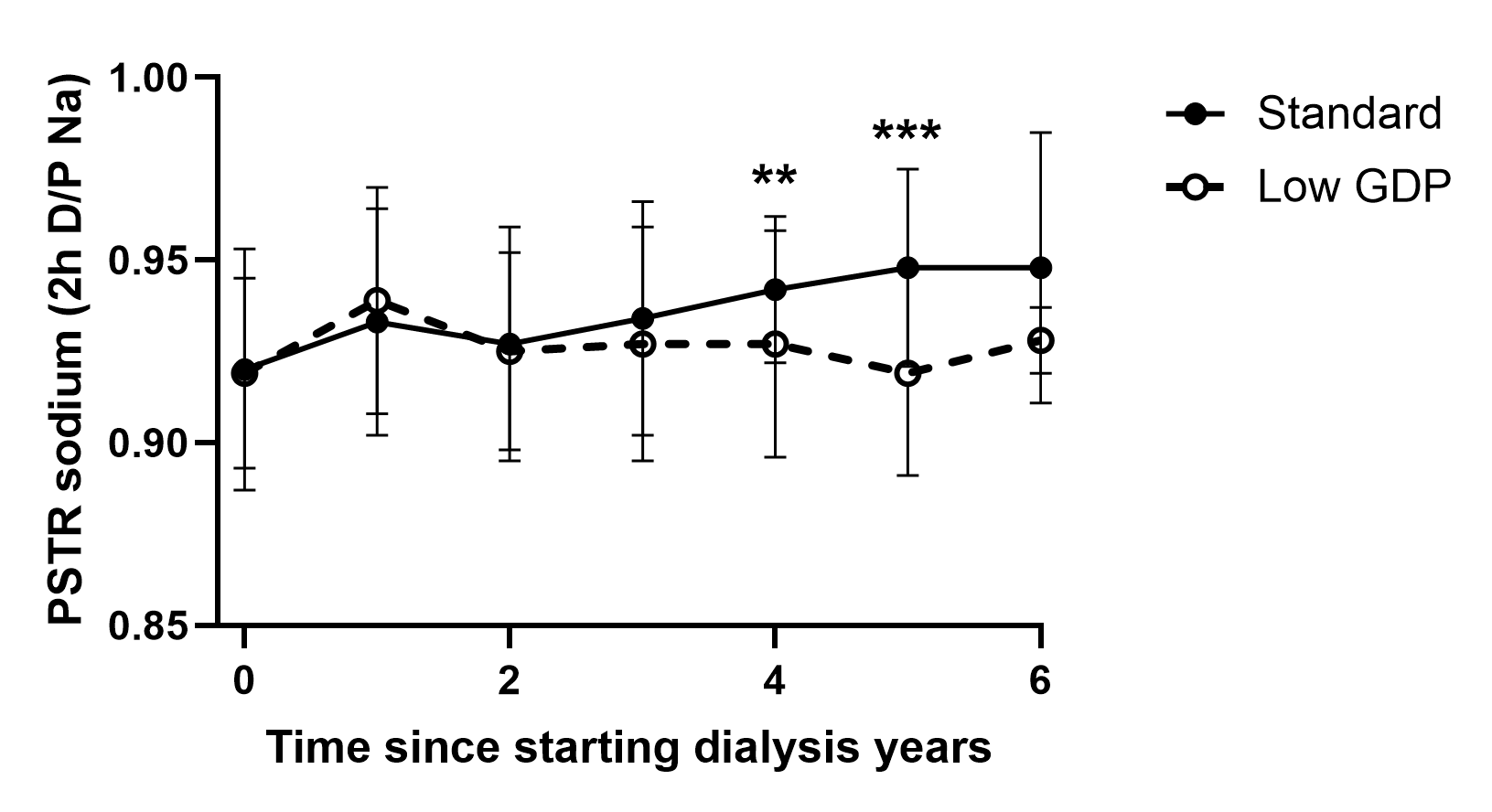

Supplement: Supplementary file 2 — Figure S2. Changes in the 2‐h effluent dialysate to plasma serum sodium (D2h D/P Na) ratio after 2‐h using a2.0 L 22.7 g/L exchange, comparing the result obtained with r the first assessment of peritoneal membrane function (PET) over time. Median with interquartile and 10%–90% ranges. **p < 0.01, ***p < 0.001 vs. first PET. [file TAP-29-471-s002.tif]
